# Educators' Perspectives on Integrating Technology Into Sexual Health Education: Implementation Study

**DOI:** 10.2196/31381

**Published:** 2022-01-12

**Authors:** Martha J Decker, Salish Harrison, Melisa Price, Abigail Gutmann-Gonzalez, Jennifer Yarger, Rachel Tenney

**Affiliations:** 1 Department of Epidemiology and Biostatistics Philip R. Lee Institute for Health Policy Studies University of California, San Francisco San Francisco, CA United States; 2 Philip R. Lee Institute for Health Policy Studies, Bixby Center for Global Reproductive Health University of California, San Francisco San Francisco, CA United States; 3 Clinical and Translational Science Center University of California, Davis Davis, CA United States; 4 School of Medicine University of California, San Francisco San Francisco, CA United States

**Keywords:** adolescent, sex education, technology, mobile app, implementation, California, health educator

## Abstract

**Background:**

In the last decade, the use of technology-based sexual health education has increased. Multiple studies have shown the feasibility of technology-based interventions, while a subset has also shown efficacy in improving youths’ sexual health outcomes such as increased condom use and knowledge. However, little is known about health educators’ experiences in integrating technology to augment sexual health curricula.

**Objective:**

The purpose of this study was to assess the perceptions and experiences of health educators regarding the incorporation of technology into a sexual health education program designed for underserved youth in Fresno County, California, and to identify facilitators and challenges to incorporating technology into the in-person curriculum.

**Methods:**

This implementation study used data collected as part of a cluster randomized controlled trial to evaluate In the Know (ITK), an in-person sexual health education curriculum that includes technology-based content, such as a resource locator, videos, and games, which can be accessed through a mobile app or website. Data from implementation logs from each cohort (n=51) and annual interviews (n=8) with health educators were analyzed to assess the health educators’ experiences using the technology and adaptations made during the implementation.

**Results:**

The health educators reported that technological issues affected implementation to some degree: 87% of the time in the first year, which decreased to 47% in the third year as health educators’ familiarity with the app increased and functionality improved. Technology issues were also more common in non–school settings. Successes and challenges in 3 domains emerged: managing technology, usability of the ITK app, and youth engagement. The health educators generally had positive comments about the app and youth engagement with the technology-based content and activities; however, they also noted certain barriers to adolescents’ use of the mobile app including limited data storage and battery life on mobile phones.

**Conclusions:**

Health educators require training and support to optimize technology as a resource for engaging with youth and providing sensitive information. Although technology is often presented as a solution to reach underserved populations, educational programs should consider the technological needs and limitations of the participants, educators, and settings.

**International Registered Report Identifier (IRRID):**

RR2-10.2196/18060

## Introduction

The use of technology-based sexual health education programs aimed at reducing sexually transmitted infections and unplanned adolescent pregnancy has increased over the last decade. Teaching with technology can be defined as any type of educational process that incorporates digital technology tools such as television, computers, tablets, smartphones, mobile apps, online educational games, or online collaborative learning environments to advance student learning [1].

The use of digital technologies in sexual health education programs has increased for multiple reasons. Some data suggest that youth access to the internet and web-based content has become nearly ubiquitous. A Pew Research report showed that 95% of adolescents aged 13-17 years had access to a smartphone in 2018 with almost 45% reporting being online on a “near-constant” basis and 90% going online multiple times per day [2]. Using technology for entertainment and information seeking may be particularly appealing in adolescence, and technology may also help to reinforce adolescent developmental growth through exploration and social connection [3]. Digital technology may also help alleviate student and teacher embarrassment, which is common when discussing sensitive subjects during sexual health classes [4]. In addition, technological tools may be able to reach and educate marginalized youth who lack access to quality and inclusive sexual health education in their schools [5,6]. However, recent research shows that a digital divide persists even among young people [7]. For example, in 2019, low-income adolescents were less likely to own laptops and smartphones than high-income adolescents (36% vs 54% and 74% vs 89%, respectively) [8].

Prior research has demonstrated that youth have favorable opinions of technology-based sexual and reproductive health interventions [9-12]. Some studies also have shown that interventions that incorporate technology were effective in improving youths’ sexual health outcomes, such as condom use, abstinence, sexual health knowledge, and safer sex norms [13-15]. However, a previous review of sexual health education apps found that most lacked comprehensive sexual health content and had limited interactivity, highlighting the unmet potential for this type of platform [16].

Despite this increase in digital sexual health interventions, little is known about health educators’ experiences delivering sexual health interventions that incorporate technology-based components. Previous research on technology in general educational programming found that health educators’ lack of confidence and perceived value of the technology can be barriers to integration [17,18]. One implementation evaluation of an online sexual health program in the Netherlands reported that while teachers appreciated the interactive content, they often needed to adapt the materials based on classroom dynamics, and some found transitioning between web-based and classroom teaching challenging [9]. Coaches in a sports-based HIV program in South Africa, which included text messages as part of the intervention, identified students’ shortage of cellular data as the primary challenge [19]. With the growing interest in online and technological approaches to education, it is critical to learn from the experiences of health educators in incorporating technology to ensure that digital content is a viable resource for engaging with youth and improving sexual health knowledge and behavioral outcomes.

The purpose of this study was to assess the perceptions and experiences of health educators regarding the integration of technology into a sexual health education program called In the Know (ITK) and to identify facilitators and challenges to incorporating technology into the in-person curriculum. These results can help future program developers and health educators anticipate and mitigate common issues with technological integration and promote best practices.

## Methods

### Intervention Overview

ITK was developed by and for adolescents aged 13-19 with a goal of increasing use of contraceptive and clinical health services [20]. Adolescents representing diverse priority populations engaged in a user-centered design process to help create the program’s content and digital components [21,22]. The curriculum is based on a positive youth development approach, which promotes personal strengths and healthy development through supportive opportunities and experiences [23,24].

The program was developed to be inclusive and to address the needs of homeless and unstably housed youth; youth of color; and lesbian, gay, bisexual, transgender, and queer or questioning (LGBTQ+) youth. ITK combines 6 hours of in-person sexual health education with technology-based content to provide the skills, information, and resources necessary to improve the sexual and reproductive health and overall well-being of adolescents.

The intervention is divided into three modules: (1) sexual health and contraceptive use; (2) healthy relationships; and (3) educational and career success. Health educators incorporated different technology-based components in each module, such as videos, online goal setting, career opportunities, and geo-location of local services. Some content was “gamified” using Kahoot, a game-based learning platform, and app-based quizzes and activities to earn points. Health educators concluded each module with a guided activity on the app and then assigned a task for the youth to complete outside of class. Youth could also sign up to receive text message reminders of key content and personal goals. These tools as well as additional resources and quizzes were available through a downloadable app or website, enabling youth to access the information outside of the in-person sessions. Health educators provided tablets with the app previously installed for use during the in-person sessions, though the participants also were encouraged to download the app on their mobile phones. The health educators helped to troubleshoot any technical issues youth were experiencing with the app.

The health educators received training on the curriculum, classroom management, and the technological components prior to implementation. This included in-person trainings and “teach backs” as well as shorter refresher trainings throughout the implementation period. The health educators reported technical issues about the app to the website developers. In addition, the developers updated features and replaced broken links over the course of the program. The researchers, health educators, and the app developers met biweekly to discuss any implementation challenges and adaptations.

### Setting and Participants

ITK was implemented in 51 cohorts (groups) with 559 youth at 36 youth-serving agencies representing a variety of settings where youth receive services or activities in Fresno County, California. The health educators traveled to the sites of the participating agencies for implementation, which included school and after-school settings, employment and training sites, youth development centers, clubs, foster care sites, housing authorities, tribal agencies, and LGBTQ+ programs. The majority of participating youth were Latino (70% [n=381]) with a mean age of 15.5 years (SD=2.07). Almost all of the participants owned or shared a smartphone (89% [n=480]), and 86% (n=469) had access to the internet in their homes.

Over the 3 years of implementation, a total of 6 health educators implemented ITK, with an average of 3 health educators per year. The health educators had a range of educational backgrounds, prior teaching, or training experience, and were comfortable with technology. This varied from 1 educator with over 6 years teaching comprehensive sexual health education to 2 educators with no prior experience in sexual or reproductive health and limited familiarity with technology; 2 other health educators had at least 2 years of experience implementing sexual health education in similar settings. Moreover, 2 health educators were male, and all lived in Fresno County.

### Data Collection

This implementation study used data collected as part of the cluster randomized controlled trial [20]. Due to the complexity of the intervention being evaluated, a better understanding of the contextual factors, including the technology and in-person implementation, can help to improve future interventions and interpret the intervention’s outcomes [25]. Process data from implementation logs and annual interviews with health educators were collected to assess fidelity to the intervention and to promote ongoing quality improvement.

#### Implementation Logs

Health educators completed an implementation log after delivering the program to each cohort. A cohort is a distinct group of participants receiving ITK at a specific time, such as a classroom of students. Each log consisted of 6 main sections: physical space, teaching methods, learning environment, youth participation, classroom management, and technology. The health educators were encouraged to comment on any contextual factors or circumstances that facilitated or hindered program delivery for specific activities or for the entire cohort. Each log also included a closed-ended question, “Thinking about what happened across all of the modules of this cohort, how often did technology issues impact implementation?” with the response options being all, most, some, or none of the time. At the end of each cohort, the health educator uploaded the completed log to Box, a secure online file management system. The researchers reviewed the implementation logs for completeness and accuracy after submission and debriefed with the implementing health educator.

#### Health Educator Interviews

The researchers conducted annual interviews near the end of each school year with the health educators for 3 years. Due to staffing changes over that time, 2-3 health educators were interviewed each year, with 2 of the health educators interviewed twice. Topic areas included implementation experiences, youth reactions, perspectives on the digital technology components, and recommendations. The interviews were conducted in a private office and averaged 53 minutes in length. All interviews were audio recorded and transcribed verbatim. Health educators received a US $20 gift card in appreciation of their time.

### Analysis

This study used a modified form of grounded theory in which a set of potential concepts were identified and coded, and additional themes were inductively identified from the data [26]. The qualitative analysis was guided by structural themes based on key areas of research interest, such as technology use, emerging themes from the review of transcripts, and the open-ended responses in the implementation logs [27]. This mixed coding system combined an initial list of codes using the main research questions and additional codes that were added based on further review [28].

One researcher coded all transcripts, while another double-coded a subset and reviewed coding for intercoder consistency. The coded interviews had an interreliability score of 0.80. The research team met regularly to review the coding process, clarify codes, and update the codebook. As needed, the researchers reviewed the quotes that were coded differently and jointly agreed to their coding. The codes were analyzed for patterns, with relevant themes extracted. The findings were also compared by year and by health educator to assess if experiences varied over time or by person. The qualitative coding was conducted using Dedoose, version 8.0.35 (SocioCultural Research Consultants, LLC) [29].

The responses to the closed-ended question regarding the frequency of technology-related interruptions were extracted and summarized using Stata 16 (Stata Corp). We used the Fisher exact test to compare the responses by whether the cohort received the program in the first year of implementation and in a school setting. One-sided *P* values are reported.

## Results

### Technology Issues During Implementation

**Figure 1:**
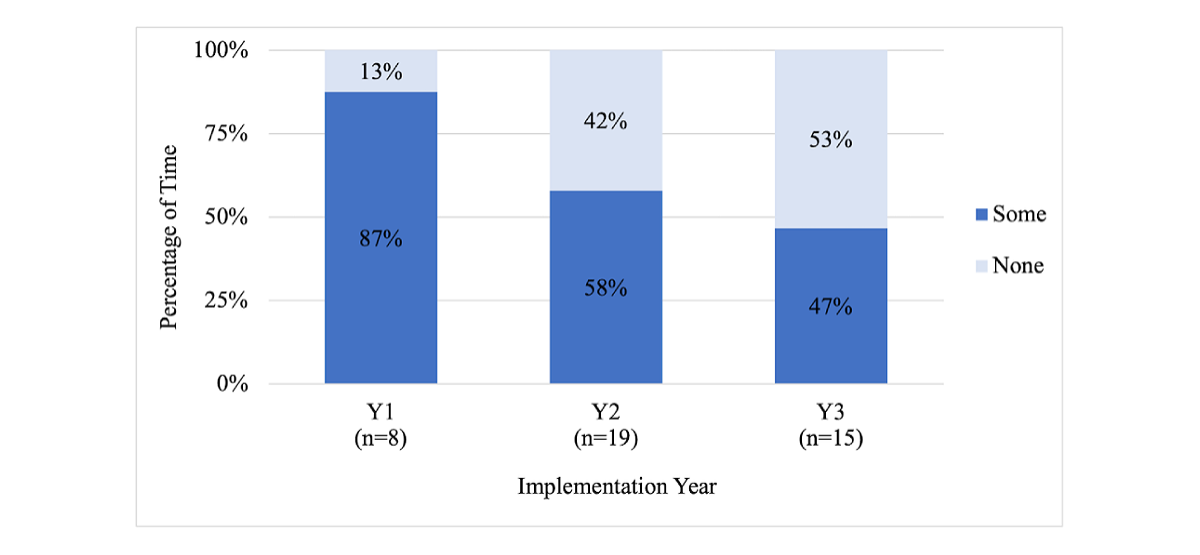
Percentage of time when implementation was affected by technology, by year (n=42).

Implementation logs were completed for all 51 in-person sessions of ITK conducted between October 2017 and February 2020. During this time, 8 interviews were completed with the health educators. During the first year of implementation, the health educators reported that technology issues affected implementation to some degree of the time in 7 out of 8 cohorts (87%) with that amount decreasing over the next 2 years, to 11 cohorts out of 19 (58%) and 7 cohorts out of 15 (47%), respectively (Figure 1; note that there were missing responses from 9 implementation logs in year 1 since one question on technology issues was added later). When calculated with the Fisher exact test, the difference between the first year and subsequent years was only marginally significant (*P*=.08) due to the small sample size. The cohorts implemented in non–school settings such as in group homes or community-based organizations were much more likely to have technology issues than those in school settings; 14 out of the 19 (74%) non–school setting cohorts experienced technology issues compared to 11 out of 23 (48%) of cohorts implemented in a school setting (*P*=.05).

Successes and challenges emerged in 3 key domains: managing technology, usability of the ITK app, and youth engagement. Managing technology included issues related to meeting the technological requirements and administrative needs for implementation during the in-person ITK sessions, such as device compatibility, internet access, and availability of necessary technology hardware. The topics related to the ITK app’s usability were those specific to the content and functionality of the app, the integration of the app into the in-person ITK curriculum, and the participants’ use of the app. Youth engagement referred to how integrating technology into the curriculum affected the participants’ focus and engagement during in-person implementation. Note that many of the issues overlapped; for example, challenges with internet connectivity limited access to the app’s content, which then affected youth engagement. Table 1 summarizes the successes and challenges within these 3 domains.

**Table 1:** Key successes and challenges of integrating technology into in-person sexual health education, by domain.

Domain	Successes	Challenges
Managing technology	Implementation sites with audiovisual projection devices present (eg, TV, projector, speakers) Bringing mobile Wi-Fi hotspot Providing tablets for classroom use	Significant preparation time required Packing tablets, Wi-Fi packs Ensuring all devices were charged and functional Implementation site lacked necessary hardware Technology issues caused delays or omission of instruction Tablets freezing and needing to restart Internet connectivity issues Inability to connect to the Internet led to alternative instructional methods Use of hard copies instead of digital content
ITK (In the Know) app usability	Positive response to online content and resources App used as reference for local services	Certain smartphone operating systems not compatible with the app Specific functionalities within the app repeatedly crashed Broken links within the app Barriers to downloading the app on smartphone included lack of data, shared phone, limited battery life Reluctance to download the app Youth forgot email address or password needed to access the app Youth did not use the app after the in-person sessions
Youth engagement	Use of tablets increased youth engagement Certain digital content resonated well with youth	Youth playing on electronic devices led to distractions

### Managing Technology

Implementation of ITK involved managing multiple technological devices and administrative requirements such as connecting program tablets and participant smartphones to the internet and projecting digital content on a screen. The health educators noted that implementation was easier in sites that had the necessary audiovisual projecting devices, such as a monitor and projector and internet access. The health educators consistently reported the challenges associated with using technology during implementation, though this decreased in frequency each year. An inability to access high-speed wireless internet was the most commonly described technology issue reported. The health educators adapted to this issue by bringing their own mobile Wi-Fi hotspots to the sites. Other common technology issues included tablets freezing or crashing during use, the lack of audiovisual projecting devices, broken web links to external online content, and youth forgetting log-in information. One health educator described common experiences with the technology:

As much as you rely on it and as great as it is, sometimes the links aren’t working, the buttons aren’t working, the screen goes blank, and you’re pressing the button and nothing’s working. Then you have to restart it.

Interview, Year 1

These technology-related issues caused delays and required health educators to adapt how they delivered the program, both ad hoc and while preparing for future implementation sessions. One health educator described an example of an ad hoc adaptation as such:

I had to use downloaded version of materials due to internet connections. Students were really excited for Kahoot [online learning platform] but, unfortunately, the game was not showing the possible answer to the students, and they could not participate the way it is usually played. I ended up reading the questions out loud and had the youth raise hands when the answer sounded correct.

Log, Year 2

The health educators provided tablets for participants to access the app if they did not download it on their personal phones. While this increased access to the materials, managing the tablets required significant planning and preparation time, as health educators needed to ensure that all electronic devices were charged and functioning properly. One health educator gave the following explanation:

We always get our materials ready… very important is coming to make sure all the tablets are charged… so we don't have any delays the next day.

Interview, Year 3

### ITK App Usability

The health educators generally had positive comments about the content of the ITK app, particularly the interactive map linking youth to resources within their community. The health educators used these online resources with the participants to identify community clinics, counseling services, and help lines for youth and families experiencing violence. One health educator described the benefits of having resources consolidated on the app:

I really do love the resources of [the app]. I always let the youth know, like, “Hey, in the app that we talked about, you know, if you have any other questions, it'd be really great for you to go on the app and you can find basically anything... There's numbers, there's addresses...” Because a lot of, some of, them do have questions that sometimes I don't know how to answer right off the bat. So I say, “Hey look at the app,” so that's really great.

Interview, Year 3

While youth could access the ITK website on a tablet during class, ITK originally was designed as an app for youth to download on their phones for later access. However, youth often faced challenges in using the mobile app including limited storage or data, limited battery life, lack of a personal cell phone number, difficulty remembering the required password, and sharing the phone with other family members. One health educator noted the following experience:

You have another group of kids that have phones, but there's always a reason why they don't want to download the app. They don't want to, they don't have space on their phone, their phone is like some crazy off-brand they can't find it. They don't have battery, phone is totally cracked, something about service, something about problems downloading the app. I don't know, it's different every time.

Interview, Year 2

Youth also expressed reluctance to download the app due to confusion about the purpose and utility of the app as well as its connection to the in-person curriculum, resulting in a limited use of the app outside of the in-person sessions. Additionally, some youth did not have access to a smartphone at all, which not only prevented them from accessing the app outside of the in-person sessions, but also contributed to the participants feeling left out of the program. A health educator described the experience of 1 youth who was homeless as such:

One participant mentioned that she felt like she was being discriminated against because she didn’t have a cell phone....

Interview, Year 1

Health educators also noted a lack of integration between the in-person elements of the curriculum and the ITK app. Because many of the ITK app features and activities were explained at the end of the modules, health educators commented on the difficulties of transitioning between the in-person curriculum and technology-based activities. One health educator stated the following:

I wish there was more involvement of the app in the actual curriculum… Like, yes, there is the whole, you know, app introduction for each module after their curriculum. But I wish it was something that we can use tied into our curriculum... It just kind of seems like the little, little side perk to the class—which it is, it is a perk, because like the other youth, who have not participated in the program, don't get to experience the app or get to have the information on the app. But I think it would still really help if we can actually use the app for facilitating, and that the youth can go back on the app and look through things that we've talked about already, or stuff like that.

Interview, Year 3

### Youth Engagement

Overall, the health educators reported that youth were engaged and interested in the curriculum. They stated that participation and engagement increased among the youth when playing games with Kahoot, an online learning platform that allowed educators to gamify content delivery. One health educator explained it as such:

Oh, Kahoot. When it's working, it works great. Like when it's working, it's probably like the one thing that the youth get excited about, maybe because they already know what it is and they get to play it at school already. So they think right away, like, “Oh, yes, it's a game!”

Interview, Year 3

Youth also responded particularly well to activities utilizing the O*Net OnLine website, a free online career exploration tool. However, health educators also noted that youth preferred participatory activities in general, whether technology-based activities and games or in-person activities such as role plays compared to lecture-based activities. One health educator described their experience as follows:

Sometimes we're not using the tablets or we're not doing like any kind of more of a group discussion. Like when there's listening in or something, or when I'm just asking them questions, it's really hard to, it's like school. It's like, okay, raise your hand or something like that. That's where I start to lose them.

Interview, Year 3

Despite fostering interest and engagement, in some instances, the presence of electronic devices was distracting for some youth. One health educator described a common experience in an implementation log as follows:

Some youths had earphones plugged in the tablets, played games, or even took photos of themselves during the class time. Facilitators would walk around the room to ask the youth to stop playing with the tablets while a facilitator was presenting. Although facilitators had to tell the youth from time to time to stop being on the tablets, facilitators did their best to move the class along with fewer distractions.

Log, Year 1

Another common youth engagement issue was the need to contextualize or personalize content for the participants. On almost every implementation log, the health educators noted instances where they had to reframe content or add explanations. For example, 1 health educator noted their role in providing supplemental information regarding a video on the biology of conception and pregnancy:

Youth did not seem to understand the video as far as the feedback that we got after when trying to discuss. Facilitator replayed the video and broke it down into different wording with each section.

Log, Year 3

## Discussion

### Principal Findings

These findings illustrate some of the successes and challenges of integrating digital technology into an in-person sexual health education program from the critical perspective of health educators. As previous studies found, health educators commonly reported that technological issues such as connectivity and device compatibility affected implementation, which were not unique to sexual health education [9,17,18]. However, technological issues became less frequent over time, likely for 3 reasons. First, health educators gained experience and confidence in addressing common technological challenges, including making innovative adaptations or finding alternatives when technology malfunctioned. Second, additional training may have led to greater familiarity and comfort with the myriad of platforms and implementation strategies. Third, health educators provided ongoing feedback to the developers, which resulted in changes to certain technology features and problem resolution. The decline in technological issues demonstrates the importance of ongoing and iterative quality improvement processes and the need for sustained engagement by the app development team in any technology-based health education intervention. It also illustrates the need to ensure that health educators are comfortable and confident in using technology, either through prior experience or through training.

Despite the implementation challenges, the health educators held positive views about the value that technology added to the in-person education, particularly in engaging youth with the material. Overall, youth tended to be more involved when they actively interacted with the content, whether through the technology-based components or in-person activities. Technology may be one of many tools that can increase the interactivity of curricular content [12]. A review of a variety of computer-based technologies found that digital games had the most evidence supporting their use to increase student engagement [30]. Game-based activities were successful, supporting the evidence that well-designed gamification can increase student engagement and motivation, and demonstrating the potential for gamification of educational content [31,32]. While the digital content was generally well received by youth, health educators also noted that the technology-based activities were not fully integrated into the curriculum. This was similar to the findings by another study of an online sexual health education course where some teachers reported difficulties transitioning between web-based and in-person activities [9].

Although adolescents have widely adopted technology, our findings are reflective of research showing ongoing disparities in technology access and use at the individual, community, and institutional level [7,33]. While ITK was designed for youth in underserved settings including foster care and shelters, health educators were more likely to encounter technology issues such as lack of Wi-Fi and other hardware in non–school settings. This made the implementation of the technology components of the program more challenging [34]. Additionally, while most participants had phones, some had limited storage or shared the phone with other family members, making them less inclined to download or keep an app, particularly one that stored sensitive information. By contrast, other studies have found that youth appreciate the anonymity available through technology-based sexual health interventions [34].

While technology can enhance youth engagement and comprehension, this study highlighted the critical role of health educators who secure the hardware necessary for implementation, adapt the curriculum when technology fails, and contextualize and personalize digital content to meet the unique needs of the youth they serve. Other studies have demonstrated the importance of staff training, confidence, and self-efficacy for the success of efforts to integrate mobile technology into education [35,36]. Beyond technological competence, health educators also need the core capabilities in knowledge and skills to deliver effective, inclusive, and appropriate sexual health education, particularly when discussing sensitive sexual and reproductive health topics [37].

### Limitations

A few limitations should be noted. The implementation log data is self-reported, so health educators may have underreported issues or interpreted a situation differently. However, these results also were consistent with annual interviews with the health educators. This study did not assess the prior experience or comfort level of the health educators with technology. In addition, because the ITK app changed over time in response to feedback and updates, some of the technical components or issues may have been resolved over time or varied by time period.

### Conclusion

As more sexual health educational programs incorporate technology, they should consider the specific role and use of technological components from both a pedagogical and logistical standpoint. Developers should engage with youth and health educators when designing health curricula and apps to ensure that the content is integrated and promotes youth learning and engagement. App developers need to invest in usability testing and a system for reporting issues throughout implementation and iteratively update the product based on that feedback. Similarly, developers and organizations need to ensure that health educators have the training, confidence, and support necessary for successful implementation, including the curricular content, classroom management skills, and necessary technology.

Although technology is often presented as a solution to reach underserved populations, that premise is not yet fully realized. Educational programs considering the adoption or integration of technology should assess the potential needs and technological capacity of the participants and settings.

## Abbreviations

ITK: In the Know
LGBTQ: Lesbian, gay, bisexual, transgender, and queer or questioning
